# S32. ANXIETY IN THE DEVELOPMENT OF PSYCHOTIC EXPERIENCES IN CHILDREN AND ADOLESCENTS: A SYSTEMATIC REVIEW

**DOI:** 10.1093/schbul/sby018.819

**Published:** 2018-04-01

**Authors:** Jessica Bird, Felicity Waite, Daniel Freeman

**Affiliations:** University of Oxford

## Abstract

**Background:**

Although traditionally separated in psychiatry, the overlap between psychosis and neurosis is becoming clear. It has been argued that emotional processes are not only a core component of psychotic experiences (PEs), but also have a causal role in symptom development. In particular, increasing evidence has highlighted anxiety may be especially important in the emergence of psychotic experiences. As the time when psychotic symptoms first emerge, understanding how anxiety influences their development in childhood and adolescence will inform early intervention. In this systematic review, we investigate the role of anxiety across the spectra of psychotic experiences in children and young people. We examine the available evidence whilst aiming to answer the questions of whether anxiety and psychotic experiences co-occur in young people and if anxiety plays a contributory causal role.

**Methods:**

A systematic literature search was conducted on PsychINFO, Medline, EMBASE, and Scopus (updated October 2017). Papers published in peer-reviewed journals were identified that investigated the relationship between anxiety and PEs in children and adolescents within both non-clinical and clinical populations. Twenty-three papers met inclusion criteria. The RTI item bank assessment framework for observational studies was used to evaluate the precision and risk of bias of each individual study.

**Results:**

The findings of this review demonstrated clear evidence for a consistent relationship between anxiety and psychotic experiences in non-clinical populations of young people, which generally displayed the highest quality of evidence. Although of overall lower quality, this relationship was also evident in clinical populations of young people with mental health problems other than psychosis. Evidence for the role of anxiety in those at clinical risk high for psychosis or diagnosed with psychosis was less conclusive, with very few studies, of variable quality, identified. These studies provided mixed findings, with some evidence for a co-occurrence between anxiety and psychosis risk/disorder, but no clear evidence of a causal role for anxiety in the transition to psychotic disorder. The size of the associations with anxiety appeared to vary by psychotic experience, with moderate evidence for hallucinations and little evidence grandiosity or anhedonia. Paranoia has the strongest evidence for a relationship with anxiety. Most studies were cross-sectional with few longitudinal studies preventing clear conclusions on a causal role of anxiety.

**Discussion:**

Evidence suggests that anxiety and psychotic experiences may co-occur in young people. This relationship appears to be transdiagnostic and potentially specific to individual psychotic experiences. As a result, more studies focusing on specific psychotic symptoms and psychological mechanisms are needed. However, research must move beyond cross-sectional associations and test the potential contributory causal role of anxiety using prospective, experimental, and interventionist designs. Despite the unknown direction of causality, the notable associations of psychotic experiences and anxiety in child mental health services indicate that this relationship may be of clinical importance. Establishing higher quality research in clinical settings is essential. Testing the effect of treating anxiety on the severity of psychotic symptoms will inform early intervention.

